# Gastroprotective Value of Berries: Evidences from Methanolic Extracts of *Morus nigra* and *Rubus niveus* Fruits

**DOI:** 10.1155/2017/7089697

**Published:** 2017-09-20

**Authors:** Luciane Angela Nottar Nesello, Maria Luisa Maes Lima Beleza, Marihá Mariot, Luísa Nathália Bolda Mariano, Priscila de Souza, Adriana Campos, Valdir Cechinel-Filho, Sérgio Faloni Andrade, Luísa Mota da Silva

**Affiliations:** ^1^Núcleo de Investigações Químico-Farmacêuticas (NIQFAR), Universidade do Vale do Itajaí (UNIVALI), Rua Uruguai, 458, Centro, 88302-202 Itajaí, SC, Brazil; ^2^Graduação em Biomedicina, UNIVALI, Rua Uruguai, 458, Centro, 88302-202 Itajaí, SC, Brazil; ^3^Programa de Pós-Graduação em Ciências Farmacêuticas, UNIVALI, Rua Uruguai, 458, Centro, 88302-202 Itajaí, SC, Brazil

## Abstract

This study evaluated the gastroprotective value of the methanol extracts from fruits of *Morus nigra* L. (black mulberry (MEMN)) and *Rubus niveus* Thunb (raspberry (MERN)). The total phenolic compounds and flavonoids were measured, as well as the *in vitro* 2,2-diphenyl-1-picrylhydrazyl (DPPH) free radical scavenger activity. The gastroprotective effects of the extracts against 60% ethanol/0.3 M HCl were evaluated in mice. After that, the lipid hydroperoxides and reduced glutathione levels at ulcerated tissue were determined. The effects of extracts on H^+^/K^+^-ATPase activity were also verified. The extracts exhibited high contents of polyphenols; however, MERN presented 1.5-fold higher levels. The presence of flavonoids also was confirmed. In addition, MEMN (IC_50_ = 13.74 *μ*g/mL) and MERN (IC_50_ = 14.97 *μ*g/mL) scavenged DPPH radical. The MEMN reduced the ulcer area only at 300 mg/kg (p.o.) by 64.06%. Interestingly, MERN decreased the ulcer area in a superior potency (ED_50_ = 20.88 mg/kg), reducing the ulcer area by 81.86% at 300 mg/kg, and increased the gastric mucin levels. The antioxidant effects of extracts were evidenced by reduced lipoperoxides and increased reduction of glutathione amount in the gastric mucosa. However, MEMN or MERN did not change the H^+^/K^+^-ATPase activity. These results confirm that *M. nigra* and *R. niveus* are berries with a gastroprotective value by strengthening of gastric protective factors.

## 1. Introduction

Gastric ulcer is a very frequent disease in the clinical practice and a challenge in the gastroenterology research [1–3]. This pathology is characterized by rupture of mucosal integrity in consequence of an imbalance between aggressive factors [acid gastric secretion, pepsin activity, and reactive oxygen species (ROS)] and the endogenous protective elements (mucus barrier, bicarbonate secretion, and adequate blood flow) of the gastric mucosa [4]. In addition, the occurrence of the gastric ulcer may be related to exogenous factors such as inadequate diet [5], alcohol consumption [6], prolonged use of nonsteroidal anti-inflammatory drugs (NSAIDs) [7], and *Helicobacter pylori* infection [8].

Currently, the antiulcer treatment can be performed with antacid drugs, such as proton pump inhibitors (PPIs) or antagonists of the type 2 histamine receptors. However, this therapy produces serious adverse effects, including osteoporotic fracture; renal damage; infection (pneumonia and *Clostridium difficile* infection); rhabdomyolysis; deficiencies of vitamin B_12_, magnesium, and iron; anemia; and thrombocytopenia [9], and is being associated with poor ulcer healing quality and in turn ulcer recurrence [10]. Therefore, alternative antiulcer therapies are required, and therapeutic resources from diet can be a relevant tool in this search.

The berries from the *Rubus* genus (Rosaceae) are distributed worldwide [11], whereas the *Morus* genus (Moraceae) is found from temperate to subtropical regions [12]. The bioactive effects of these berries have been commonly related to their phenolic compounds [such as phenolic acids, flavonoids (anthocyanins and flavonols), and tannins] and ascorbic acid contents [13]. Therefore, the recognition of these fruits as promising functional foods and their value as phytotherapics is growing around the world. Indeed, based on the folk medicine, the *Morus* genus is listed by the Brazilian government as a plant species with promising potential in human health improvement [14].

In view of the above, the present study evaluated the gastroprotective activity of extracts obtained from fruits of the *Morus nigra* L. (black mulberries) and *Rubus niveus* Thunb (raspberries), assessing their antioxidant properties and effects on gastric mucin content, as well as on H^+^/K^+^-ATPase activity. In addition, the levels of phenolic compounds and flavonoids in both extracts were quantified.

## 2. Materials and Methods

### 2.1. Plant Material and Obtaining Extracts

In order to obtain the extracts, 24.0 g of *M. nigra* (black mulberries) and 24.0 g of *R. niveus* (raspberries) fruits were commercially obtained, manually chopped, and subjected to a maceration process with methanol. Each plant sample was separately macerated with methanol in a solvent : solid ratio of 10 : 1.5, at 25°C, by seven days in a sealed glassware, and protected from light [15]. After this period, the obtained materials were filtered and the solvent was removed under reduced pressure, obtaining the methanolic extract from fruits of *M. nigra* (MEMN, 1.44 g, 6.00% yield) and the methanolic extract from fruits of *R. niveus* (MERN, 1.73 g, 7.21% yield).

### 2.2. Quantification of Polyphenol and Flavonoid Content

Total phenolic compounds were quantitated using the Folin–Ciocalteu reagent [16]. The absorbance of the extract solution (100–400 *μ*g/mL) was assessed at 750 nm. The total concentration of phenolic substances was calculated using a tannic acid curve as a standard, and the results were expressed in tannic acid equivalents (TAE).

The quantification of flavonoid on extract solution (100–400 *μ*g/mL) was performed using aluminum chloride methanol solution [17]. The absorbance was read at 425 nm, and the flavonoid content was expressed as quercetin equivalents (QE) and calculated using a quercetin standard curve.

### 2.3. *In Vitro* 2,2-Diphenyl-1-picrylhydrazyl (DPPH) Free Radical Scavenger Activity

For the quantification of total antioxidant activity, the scavenger capability of MEMN (0.01–1000 *μ*g/mL) and MERN (0.01–1000 *μ*g/mL) against the stable free radical 2,2-diphenyl-1-picrylhydrazyl (DPPH, 40 *μ*g/mL) was evaluated [18]. The decrease in absorbance (570 nm) was measured after 15 min. Samples of distilled water and ascorbic acid (50 *μ*g/mL) were used as negative and positive controls, respectively.

### 2.4. Animals

Female Swiss mice (25–30 g) from the Central Animal Laboratory of the Universidade do Vale do Itajaí (UNIVALI) were housed in polypropylene cages at 22 ± 2°C under 12 h light/dark cycle with access to food and water ad libitum. The animals were deprived of food 8 hours prior to the experiments. One adult albino rabbit (*Oryctolagus cuniculus*, 2 kg) was used to determine the gastric activity of H^+^/K^+^-ATPase. All protocols were approved by the Institutional Animal Ethics Committee of the UNIVALI (CEUA/UNIVALI; approval number 005/14).

### 2.5. Acidified Ethanol-Induced Acute Gastric Ulcer

The mice were divided into different groups (*n* = 6) and pretreated orally with vehicle (water, 10 mL/kg), carbenoxolone (used as the positive control, 100 mg/kg), MEMN (30–300 mg/kg), or MERN (10–300 mg/kg); an ulcerogenic solution composed of 60% ethanol/0.3 M HCl was orally given to the mice one hour after the pretreatments [19]. One hour after the administration of the injurious agent, the animals were euthanized in CO_2_ atmosphere, and then, the stomachs were removed, opened along the greater curvature, stretched on glass plates, and digitized. The EARP image analysis software carried out the analysis of the stomachs, in order to determine the gastric ulcer area (mm^2^).

### 2.6. Histological and Histochemistry Analyses

The ethanol/HCl-ulcerated gastric tissue from all experimental groups were fixed in a solution composed of 85% alcohol, 10% formalin, and 5% acetic acid, dehydrated, diaphanized, embedded in paraffin, and sectioned (5 *μ*m). The slices were deparaffinized, and a part of histological segments were stained using the hematoxylin and eosin technique to observe histological changes in tunics of gastric tissue. The remaining portion of the deparaffinized segments was employed in histochemistry analysis to measure the amount of gastric mucin, using Schiff's Periodic Acid method [18]. The software ImageJ® was used to quantify the gastric mucin.

### 2.7. Preparation of Subcellular Fraction

The ulcerated tissues from an ethanol/HCl-induced ulcer model were homogenized with 200 mM phosphate buffer (pH 6.5), and this homogenate was used in the quantification of lipoperoxides (LOOH) and reduced glutathione (GSH) levels.

### 2.8. LOOH and GSH Measurements

The LOOH in the ethanol/HCl-ulcerated gastric mucosa was measured [20]. In microtubes, 100 *μ*L of the homogenate obtained with the ulcerated tissue was added to equal volume of methanol, mixed, and centrifuged at 9000 × g for 20 min at 4°C. After, 30 *μ*L of the supernatant was added to 270 *μ*L of reaction medium [4 mM butylated hydroxytoluene (BHT), 250 mM FeSO_4_, 25 mM H_2_SO_4_, and 100 mM xylenol orange], solubilized in methanol, and incubated for 30 minutes at 25°C. After that, the absorbance of reaction mixture was read at 560 nm and the concentration of LOOH was determined for each 1 mg of protein using the extinction coefficient of 43.6/M/cm for H_2_O_2_, cumene hydroperoxide, or butyl hydroperoxide. The results were presented as mmol/mg of tissue.

The GSH quantification was performed in 50 *μ*L of the gastric homogenate deproteinized with 40 *μ*L of 12.5% trichloroacetic acid. The samples were centrifuged for 20 min at 900 ×g at 4°C, and subsequently, a 10 *μ*L aliquot of the supernatant was added to 290 *μ*L of 0.4 M TRIS buffer solution (pH 8.9). For the reaction to be initiated, 5 *μ*L of 5,5′-dithiobis-2-nitrobenzoic acid (1 mM) was added to each sample 15 minutes before the spectrophotometric reading at 415 nm. The individual values were interpolated in a GSH standard curve, with values expressed in *μ*g/mg tissue [21].

### 2.9. H^+^/K^+^-ATPase Activity

The gastric mucosa of a rabbit was employed for the isolation of the gastric microsomal portion [22]. The gastric mucosa were collected and processed to obtain a homogenate. The H^+^/K^+^-ATPase isolation was performed by ultracentrifugation followed by a separation gradient. Then, 100 *μ*g of H^+^/K^+^-ATPase isolated was incubated with the vehicle (10% DMSO), MEMN (1–100 *μ*g/mL), MERN (1–100 *μ*g/mL), ouabain (72.8 *μ*g/mL, an inhibitor of Na^+^/K^+^-ATPase), or omeprazole (34.5 *μ*g/mL, an H^+^/K^+^-ATPase inhibitor). The incubation period was 20 min in the presence of the reaction mixture [50 mM Tris-HCl (pH 7.4), 20 mM KCl, and 2.5 mM MgCl_2_]. After this period, adenosine triphosphate was added and the samples were incubated at 37°C for 10 min. The H^+^/K^+^-ATPase activity was calculated using the molar extinction coefficient of the inorganic phosphate (iP) (*ε* = 11,000/M·cm) and expressed in *μ*M iP/mg protein/minute [23].

### 2.10. Statistical Analysis

The data were analyzed by the statistical program GraphPad Prism® 5.0 and represented as the means ± SEM, and the differences between the means were determined through one-way analysis of variance (ANOVA) followed by Bonferroni's test. In all experiments, *p* < 0.05 was adopted as significant.

## 3. Results and Discussion

Berries are important sources of a variety of bioactive compounds, which can present beneficial effects to human health [24]. Among these nutraceuticals, the polyphenols and flavonoids have justified the pharmacological effects of berries [25–30]. Most of studies on the beneficial effects of berries are focused on cardiovascular disorders, advancing age-induced oxidative stress, inflammatory responses, diverse degenerative diseases, and cancer [31–34], and few studies reported their antiulcer effects [35–37]. Therefore, the gastroprotective effects of fruits from *M. nigra* and *R. niveus* are described for the first time in this study.

Methanol is an appropriate solvent to extract polyphenols from *M. nigra* fruits [38], and for this reason, this solvent was employed to obtain the extracts used in our study. Indeed, both extracts demonstrated high contents of polyphenols and flavonoids, as demonstrated in Table 1. The recognition that the intake of dietary polyphenols in the human diet or their supplementation parallel to conventional treatment can result in a safe and effective management of peptic ulcer is growing [39]. Regarding the flavonoids, these secondary metabolites display a wide range of pharmacological properties in the gastroenterology, acting as antisecretory, cytoprotector, and antioxidant compounds [19, 40–42].

**Figure 1:**
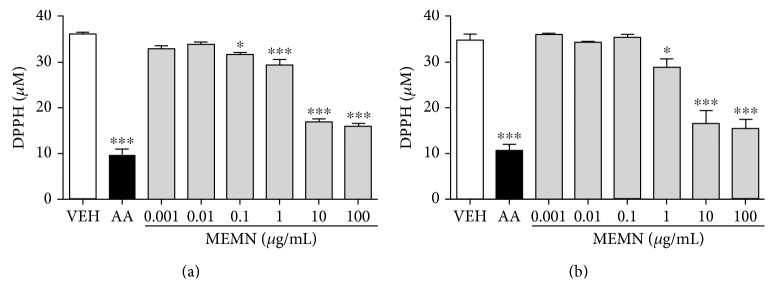
*In vitro* effects of (a) MEMN or (b) MERN (0.001–100 *μ*g/mL) to scavenge the free radical DPPH. AA: ascorbic acid (50 *μ*g/mL); VEH: vehicle (distilled water). The results are expressed as mean ± SEM, in triplicated experiments. Statistical comparison was performed using one-way analysis of variance (ANOVA) followed by Bonferroni's test. ^∗^*p* < 0.05 and ^∗∗∗^*p* < 0.001 versus vehicle group.

It is well known that phenolic compounds, which were quantified in MEMN and MERN, have high antioxidant activity [43]. Thus, the free radical scavenger activity of the extracts was measured, and the half-maximal inhibitory concentration (IC_50_) of MEMN and MERN in the DPPH assay was 13.74 *μ*g/mL (logIC_50_ = 1.138) and 14.97 *μ*g/mL (logIC_50_ = 1.170), respectively, as shown in Figures 1(a) and 1(b). Similarly, a stronger scavenger activity was described for a sugar-free methanolic extract obtained from *M. nigra* fruits [44], and an *in vitro* antioxidant activity of methanol extracts obtained from *R. niveus* was already verified [45, 46].

**Figure 2:**
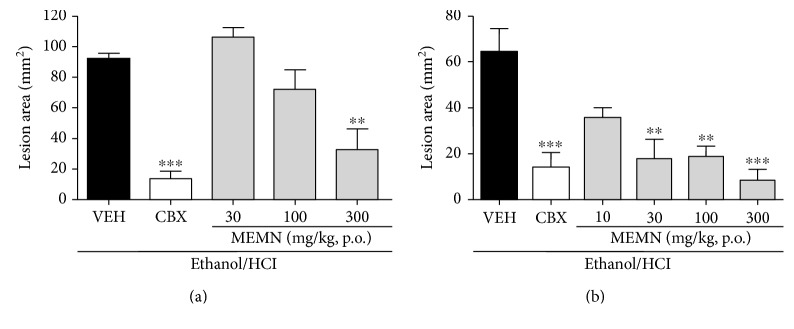
Gastroprotective effects of (a) MEMN (30–300 mg/kg, p.o.) or (b) MERN (10–300 mg/kg, p.o.) on acidified ethanol-induced gastric ulcers in mice. CBX: carbenoxolone (100 mg/kg, p.o.); VEH: vehicle (10 mL/kg, p.o.). The results are expressed as mean ± SEM (*n* = 6). Statistical comparison was performed using one-way analysis of variance **(**ANOVA) followed by Bonferroni's test. ^∗∗^*p* < 0.01 and ^∗∗∗^*p* < 0.001 versus vehicle group.

Several studies have described the involvement of oxidative stress in the pathogenesis of gastric lesions and have pointed out that redox signaling regulates several processes in the gastrointestinal epithelium [47]. In this context, the intake of antioxidants like nutraceuticals from berries may afford beneficial effects in attenuating the formation of the gastric lesions. The ethanol increases oxygen reactive species (ROS) generation, reduces the mucus protective layer, and in consequence promotes modification in gastric cellular homeostasis leading to severe damage to the gastric mucosa [48]. As shown in Figure 2(a), the MEMN presented a capacity of defense of the gastric mucosa against the acidified ethanol only at 300 mg/kg (p.o.), reducing by 64.06% the ulcer area, when compared with vehicle (92.10 ± 3.51 mm^2^). Interestingly, as shown in Figure 2(b), the MERN presented a great gastroprotective potency in this same experimental model reaching a half-maximal effective dose (ED_50_) of 20.88 mg/kg (logED_50_ = 0.057). As expected, the oral treatment with carbenoxolone (100 mg/kg), used as the positive control, also reduced the ulcer area (Figures 2(a) and 2(b)).

These findings introduce *M. nigra* and mainly *R. niveus* as a favorable source for antiulcer components, and it is important to emphasize that the potent gastroprotective effect of MERN may be related to its high polyphenol levels, since the amount of these compounds in its extract was at least 1.5-fold higher when compared to MEMN values. However, given the previous knowledge about the anti-inflammatory properties of *M. nigra* [49], the gastroprotective effect of MEMN described herein places this berry in an interesting position because most anti-inflammatory drugs do not have a good gastric tolerance and many of them are harmful agents to the gastric mucosa.

**Figure 3:**
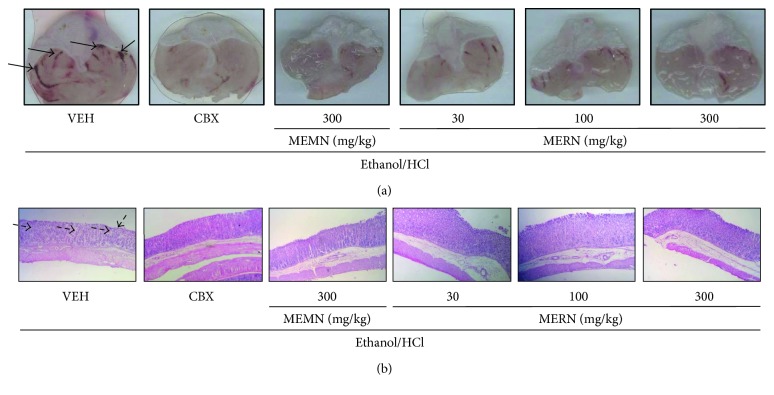
Macroscopic (a) and microscopic (b) representative images of the gastroprotective effects of MEMN and MERN on acidified ethanol-induced gastric ulcers in mice. The mice were orally treated with vehicle (water, 10 mL/kg), carbenoxolone (100 mg/kg), MEMN (300 mg/kg), or MEMN (30–300 mg/kg) 1 hour prior to acidified ethanol administration. The black arrows indicate the site of injury and the dotted black arrows show the glandular damage on the gastric mucosa. Histological section was increased by 100x.

**Figure 4:**
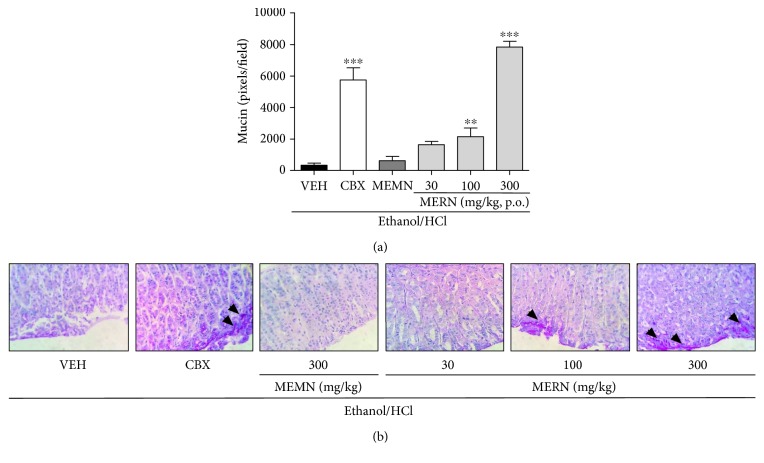
Effects of MEMN and MERN on histochemistry staining for mucin-like glycoproteins in the acidified ethanol-ulcerated gastric mucosa. (a) Representative images from groups treated with vehicle (water, 10 mL/kg), carbenoxolone (100 mg/kg), MEMN (300 mg/kg), or MERN (30–300 mg/kg). (b) Quantification of mucin staining with results expressed as mean ± SEM (*n* = 6). Statistical comparison was performed using one-way analysis of variance (ANOVA) followed by Bonferroni's test. ^∗∗^*p* < 0.01 and ^∗∗∗^*p* < 0.001 versus vehicle group. The arrows indicate the mucin-positive staining by 400x of magnification.

Representative macroscopic and microscopic images from the gastroprotective effect of both extracts are shown in Figures 3(a) and 3(b), respectively. Therefore, the gastroprotective effect of MEMN and MERN was confirmed by the decrease in the degree of epithelial damage evoked by acidified ethanol intake (Figure 3(b)). In parallel, the oral administration of MERN (100 or 300 mg/kg), but not of MEMN (300 mg/kg), also increased the mucin staining in the gastric mucosa (Figures 4(a) and 4(b)). The structural and functional integrity of the gastric tissues is maintained by the equilibrium between aggressive and protective factors; the mucus-buffer-phospholipid layer is a preepithelial barrier and remains a crucial mucosal protection [50]. Because mucins are pivotal glycoproteins present in this barrier, it is possible to infer that the gastroprotective effects of MEMN and MERN are mediated by the luminal protection favoring.  Figure 4(a) shows representative images from mucin staining from all experimental groups.

**Figure 5:**
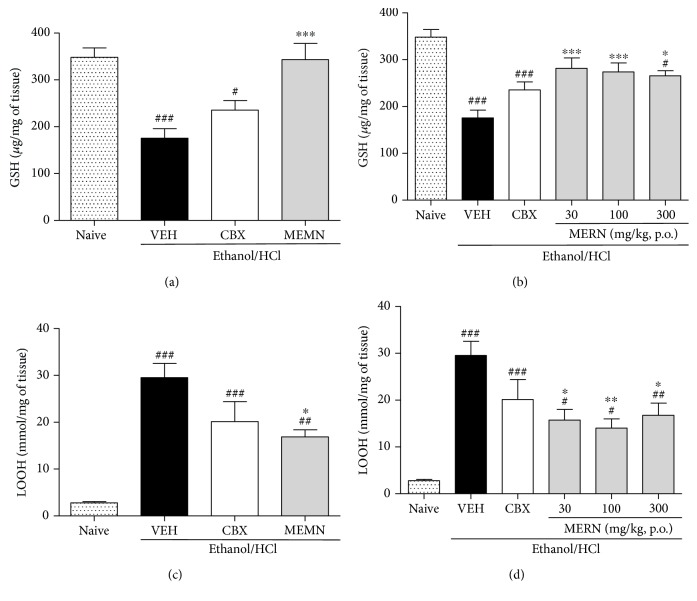
Effects of MEMN (300 mg/kg, p.o., (a) and (c)) and MERN (30–300 mg/kg, p.o., (b) and (d)) on lipoperoxides (LOOH) and reduced glutathione (GSH) levels on acidified ethanol-ulcerated tissue. The results are expressed as mean ± SEM (*n* = 6). Statistical comparison was performed using one-way analysis of variance (ANOVA) followed by Bonferroni's test. ^#^*p* < 0.05, ^#^^#^*p* < 0.01, and ^#^^#^^#^*p* < 0.001 versus nonulcerated group (naïve). ^∗^*p* < 0.05, ^∗∗^*p* < 0.01, and ^∗∗∗^*p* < 0.001 versus vehicle group (VEH, water, 10 mL/kg). CBX: carbenoxolone (100 mg/kg, p.o.).

It is well established that the gastric tissue damage promoted by ethanol is directly related to the formation of ROS, resulting in decreased levels of GSH and antioxidant enzymes. The GSH is an important cellular antioxidant and has different functions, such as the ability to scavenge ROS in parallel acting in the prevention of ROS formation [51]. As shown in Figure 5, the levels of GSH in the vehicle-treated acidified ethanol-ulcerated stomach were decreased by 50.43%. However, the treatment with MEMN (300 mg/kg, Figure 5(a)) or with MERN (30, 100, and 300 mg/kg, Figure 5(b)) was both able to prevent the GSH depletion, indicating that the restoration of the tissue homeostasis promoted by these extracts was mediated by the improvement in the antioxidant capacity of the gastric mucosa.

An important marker of oxidative stress in tissues is the lipid peroxidation, including in the gastrointestinal tract [52]. As expected, increased levels of gastric LOOH were confirmed in the ulcerated group treated with vehicle (29.57 ± 2.99 mmol/mg of tissue). On the other hand, the MEMN (300 mg/kg, Figure 5(c)) promoted the partial reduction of it, similar to the MERN at doses of 100 and 300 mg/kg (Figure 5(d)). Given these data, the hypothesis that both extracts can reduce the oxidative stress involved in the genesis of gastric injury induced by acidic ethanol is strengthened. Corroborating our findings, the inhibitory effects of a methanolic extract from fruits of *M. nigra* on the lipid peroxidation at the liver of rats were already described [53].

**Figure 6:**
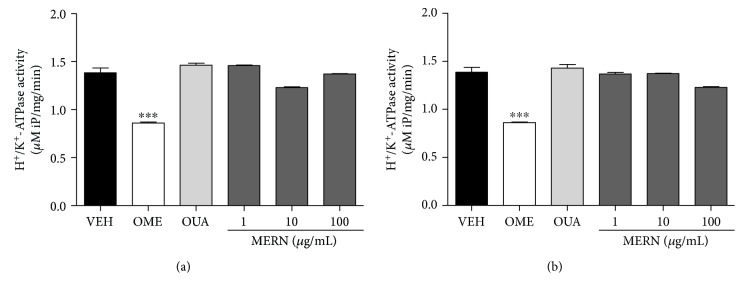
Effects of MEMN (1–100 *μ*g/mL, (a)) and MERN (1–100 *μ*g/mL, (b)) on *in vitro* H^+^/K^+^-ATPase activity. The results are expressed as mean ± SEM, in triplicated experiments. Statistical comparison was performed using one-way analysis of variance (ANOVA) followed by Bonferroni's test. ^∗∗∗^*p* < 0.001 versus vehicle group (VEH). OME = omeprazole (H^+^/K^+^-ATPase inhibitor, 34.5 *μ*g/mL). OUA = ouabain (Na^+^/K^+^-ATPase inhibitor, 72.8 *μ*g/mL).

The proton pump inhibitors (PPIs) are a pivotal class of drugs on the ulcer and gastroesophageal reflux disease therapy, although their long-term use carries the risk of several side effects that should be considered [9]. In contrast, MEMN (Figure 6(a)) or MERN (Figure 6(b)) did not change the *in vitro* H^+^/K^+^-ATPase activity, supporting the hypothesis that both extracts exert gastroprotection without altering proton pump function. Expectedly, omeprazole (34.5 *μ*g/mL), but not ouabain (72.8 *μ*g/mL), inhibited the *in vitro* H^+^/K^+^-ATPase activity by 37.54%, when compared to vehicle (1.38 ± 0.05 *μ*mol iP/mg/min). These findings reinforce that the studied berries constitute a promising source of natural products with potential complementary use in the current therapy, acting in a different way from the most conventional antiulcer drugs in clinical practice.

## 4. Conclusion

Taking together, the results from this study are promising and confirmed that the methanolic extracts obtained from the fruits of *M. nigra* or *R. niveus* concentrate on phenolic compounds, which can ensure their antioxidant and in turn gastroprotective potential.

## Figures and Tables

**Table 1:** Total phenolic compound and flavonoid amount in methanolic extract from *Rubus niveus* fruits (MENR) and in methanolic extract from *Morus nigra* fruits (MEMN).

Extract	Total phenolic compounds	Total flavonoids
MERN (*μ*g/mL)	TAE ± SEM	QE ± SEM
100	59.50 ± 2.41	0.23 ± 0.04
150	93.06 ± 1.78	0.35 ± 0.01
200	132.96 ± 7.49	0.41 ± 0.02
MEMN (*μ*g/mL)	TAE ± SEM	QE ± SEM
100	44.79 ± 1.22	0.30 ± 0.21
150	60.51 ± 6.71	0.48 ± 0.04
200	88.63 ± 6.02	0.60 ± 0.03

The results are expressed as mean ± SEM of tannic acid equivalents (TAE) and quercetin equivalents (QE) in *μ*g/mL.
